# An Evaluation of T-Regulatory Cells and Inflammatory Cytokines in Preeclampsia

**DOI:** 10.7759/cureus.43379

**Published:** 2023-08-12

**Authors:** Bharath Kumar Koppisetty, Prakruti Dash, Gautom K Saharia, Saurav Nayak, Saubhagya K Jena

**Affiliations:** 1 Biochemistry, All India Institute of Medical Sciences, Bhubaneswar, Bhubaneswar, IND; 2 Obstetrics and Gynecology, All India Institute of Medical Sciences, Bhubaneswar, Bhubaneswar, IND

**Keywords:** high-risk pregnancy, hscrp, interleukin (il)-6, tgf-β1, inflammatory cytokines, t-regulatory cells, pre-eclampsia

## Abstract

Background

Preeclampsia (PE) is a prenatal hypertension condition with unknown aetiology which is one of the leading causes of maternal morbidity and mortality, premature delivery, and foetal and neonatal mortality. T-regulatory cells (T-regs) are the specific subsets of T-lymphocytes that play a key role in the mechanisms of maternal-foetal tolerance, contributing to an effective immunological role in protecting the allogenic foetus during pregnancy and preventing pregnancy-related complications. This study evaluated the T-regs in PE and correlated the T-regs with inflammatory markers in the pathophysiology and for early diagnosis of PE.

Methods

After clearance from Institutional Ethics Committee, the participants were recruited from the Department of Obstetrics and Gynaecology. Three study groups were included a) normal reproductive age group women b) normal pregnant women c) PE pregnant women. 5 ml of venous blood was collected from each participant. Biochemical and haematological parameters estimation was done in Hospital’s central laboratory. T-regs (CD4, CD25, FOXP3) were assessed using a flow-cytometer, and inflammatory markers (TGF-β1, IL-6, hsCRP) were assessed by ELISA and Beckman Coulter autoanalyzer in the Department of Biochemistry, AIIMS, Bhubaneswar.

Results

We found that the levels of CD4+CD25+ T-regs were lower in PE than in normal pregnancy, but this difference was not statistically significant (p = 0.349). The levels of CD4+FOXP3+ T-regs in PE were significantly lower compared to both normal pregnant women (p = 0.001) and normal non-pregnant women (p = 0.001). In comparison to women with PE, the levels of TGF-β1 were significantly higher in normal non-pregnant women (p = 0.020) and were higher, although not significantly so, in normal pregnant women (p = 0.994). The levels of IL-6 in women with PE were significantly higher than in normal pregnant women (p = 0.01) and normal non-pregnant women (p = 0.048). The levels of hsCRP in women with PE were significantly higher than in normal pregnant women (p = 0.045) and were higher, but not statistically significant, compared to normal non-pregnant women (p = 0.094).

Conclusion

The results of the study, showing a decrease in T-regs and an increase in inflammatory markers like TGF-β1, IL-6, and hsCRP levels in PE, have potential implications for the early diagnosis and management of the condition. Incorporating assessments of CD4+FOXP3+ T-regs and inflammatory markers into screening protocols, along with regular prenatal care and monitoring, can aid in the timely detection and implementation of appropriate management strategies. By intervening early, the risks associated with PE can be reduced, optimizing both maternal and fetal health.

## Introduction

Preeclampsia (PE) is a common pregnancy-associated complication affecting 2-5% of pregnant women. Globally it is one of the major causes of prenatal morbidity, which raises the mortality risk from obstetric complications by up to 15%. PE primarily brings about premature births and fetal growth restriction (FGR) in the fetus, although the mother can also suffer from complications such as eclampsia and renal or liver failure. Women who are typically symptomatic and were previously normotensive are diagnosed with hypertension if their blood pressure is ≥140/90 mmHg after 20 weeks of pregnancy and if they exhibit any of the following: proteinuria of more than 300 mg in a 24-hour urine sample, maternal organ dysfunction, or uterine and placental abnormalities [1]. Although there have been advances in understanding the etiology of PE, the primary mechanism, which is likely complex, continues to remain unknown. Feto-maternal immunity, however, is one of the systems that has received the most attention. Growing literature data suggests that the development of PE is linked to a disparity between pro-inflammatory and anti-inflammatory substances, which triggers a systemic immune reaction and involves the arterial endothelium. In an uncomplicated pregnancy, NK cells, dendritic cells, and regulatory T-cells maintain immunotolerance towards spiral artery remodeling and developing fetal trophoblasts. These local microenvironmental factors work together to influence how well the mother's immune system tolerates the fetus as an allograft. Poor placentation can lead to increased shedding of the placenta, a heightened systemic inflammatory response, and eventual vascular endothelial disturbance, all of which contribute to the symptoms of PE [1]. Although proteinuria and hypertension PE can be diagnosed, there is still a tremendous need for more laboratory indicators that can predict, diagnose, or categorize risks of developing PE early in pregnancy. Currently, only a few markers, namely placental induced growth factor (PIGF), soluble fms-like tyrosine kinase-1 (sFlt-1), and pregnancy-associated plasma protein A (PAPP-A), are used to test for PE. For uniform algorithms, there were no clearly defined testing points of time or precise cut-offs. Another lacuna is the use of such markers in the general population of pregnant women, or the comparison of data with the specific population from which they were drawn is not available. Furthermore, given that this illness may impact both the mother and the baby, new markers should identify PE before the signs of hypertension appear [1].

A specialized sub-group of T-lymphocytes called T-regulatory cells (T-regs) reduces inflammation and prevents transplant rejection and autoimmune reactions [2]. T-regs inhibit maternal T cells from becoming activated against the fetal cells; this defense of the fetus against the mother's immune response has been extensively documented for both mice and humans [3]. Decreased levels of maternal T-regs may hinder the immune acceptance of the fetus, potentially leading to obstetric complications like PE, miscarriage, and preterm labor [4-5].

Inflammatory cytokines such as transforming growth factor beta-1 (TGF-β1) are required to inhibit T-cells and control inflammation, and they are crucial for the development of T-regs that induce immune tolerance [6]. According to studies, TGF-β1 is a multifunctional cytokine that plays a key role in the etiology of PE. TGF-β1 has been shown to affect endothelial cell apoptosis, embryonic growth, and development, and it has recently been recommended as one of the potential candidate genes for PE [7]. TGF-β1, considered the master regulator of T-regs, also monitors the percentage of these cells, which are essential for maintaining self-tolerance, physiological immune reactions, and mediating maternal tolerance to the fetus. Increased levels of circulating T-regs are a hallmark of normal pregnancy, in contrast to the reduced levels seen in women who experience pregnancy complications such as PE and pregnancy loss [8]. High-sensitivity C-reactive protein (hs-CRP) is produced in hepatic tissue in response to stimulation by interleukin-6 (IL-6), and it can serve as a marker for tissue injury and systemic inflammation [9]. IL-6 plays a critical role in bridging the gap between the innate and acquired immune response by promoting the specialized development of naive CD4+ T cells. It has been established that when combined with TGF-β, IL-6 is necessary for converting naive CD4+ T cells into Th17 cells. However, IL-6 can also inhibit TGF-β from inducing T-reg cell differentiation. The breakdown of immunological tolerance is mainly caused by the upregulating of the Th17/T-reg balance, resulting in pathological conditions such as autoimmunity and chronic inflammatory disorders [10]. PE is characterized by a transition towards a continuous inflammatory response and endothelial impairment. Pro-inflammatory cytokines and growth factors are the main driving cause of developing PE. At the same time, anti-inflammatory effectors such as T-regs show diminished functioning. Unfortunately, the fundamental etiology of PE has not been linked to a confirmatory mechanism. For these reasons, research regarding PE is necessary to provide cutting-edge knowledge of pathophysiology, identify new diagnostic techniques, and develop disease-targeted therapies [1]. Hence, this research was undertaken to assess the T-regs in PE. Inflammatory markers like IL-6, TGF-β1, and hs-CRP were correlated with T-regs to understand the pathophysiology and for early diagnosis of PE.

## Materials and methods

Study population

This is a cross-sectional study conducted in the Department of Obstetrics and Gynaecology, Department of Biochemistry in AIIMS, Bhubaneswar. This study included 3 groups a) Preeclampsia pregnant women (PE), b) Normal pregnant women (NP), and c) Normal non-pregnant women in the reproductive age group (NW). Those meeting the inclusion and exclusion criteria were enrolled in the study.

Inclusion Criteria

a) PE pregnant women: PE comprised a group of women matching the diagnostic criteria of the International Society for the Study of Hypertension in Pregnancy (ISSHP). The criteria include two separate blood pressure readings of ≥ 140/90 mmHg and either 300 mg of proteinuria over 24 hours or a protein dipstick analysis of +1; (b) Healthy pregnant women: pregnant women with more than 20 weeks of gestation, including both primiparous and multiparous pregnant women; and (c) normal non-pregnant women.

Exclusion Criteria

Women with (a) HELLP syndrome; (b) eclampsia; (c) diabetes mellitus; (d) other inflammatory disorders; and (e) autoimmune disorders were excluded.

Sample Size

A total of 50 participants were included in three groups: (a) PE, 20 participants; (b) NP, 20 participants; (c) Healthy NW, 10 participants.

Method

The study was conducted in the Department of Biochemistry at AIIMS, Bhubaneswar. The participants were selected from women attending the Obstetrics and Gynaecology department at AIIMS, Bhubaneswar. A total of 5 ml of venous blood was collected from each study subject. A 2 ml blood sample was collected in an EDTA vacutainer tube, and this fresh sample was used for the estimation of T-regs (CD4, CD25, and FOXP3) by flow cytometry analysis. The remaining amount was collected in a plain vacutainer tube, and the serum was separated immediately after centrifugation and stored at -20 ℃. The serum was used for the estimation of IL-6 and TGF-β1 by the sandwich enzyme-linked immunosorbent assay (ELISA) method, and hsCRP was estimated using the immunoturbidimetry method with a Beckman Coulter 5800 auto-analyzer.

Flow Cytometry

The Beckman Coulter Navios flow cytometer was used to analyze the processed samples. The computer system, attached to the flow-cytometer machine, stored the data for each cell, which was then analyzed using the inbuilt software CXP. Three types of cells were evaluated, i.e., CD3+CD4+ depicting complete T-lymphocyte population, CD4+CD25+ indicating T-regs, and CD4+FOXP3+ with FOXP3 as the absolute intracellular marker for accurate identification of T-regs. DURAClone IM Treg tubes by Beckman Coulter were used for flow cytometry analysis of T-reg markers (CD4+, CD25+, FOXP3+). Each kit contains two sets of antibody-coated tubes with 25 tubes in each set. Two sets were (a) Tube 1 for T-cell surface markers and (b) Tube 2 for T-cell intracellular markers. Flow cytometry analysis is based on the ability of a specific monoclonal antibody to bind to the antigenic determinants expressed by different T cell subpopulations. The specific surface staining of the T cell is carried out by allowing the sample to sit in DuraClone IM Treg Tube 1. To enable the labeling of intracellular determinants, PerFix nc Buffer 1 is used to fix cells in DuraClone IM Treg Tube 2, and Buffer 2 is utilized to induce permeability in the cytoplasmic and nuclear membranes of the T lymphocytes. After permeabilization, the flow cytometer measures light diffusion and cell fluorescence; it also allows for the electronic gate-based delimitation of the population of interest based on a bivariate histogram that correlates the diffusion of narrow-angle light (Forward Scatter or FS) with orthogonal light (Side Scatter or SS). TGF-β1 analysis was done using the EliKine™ Human TGF-β1 ELISA kit (96 wells), which employs a double antibody sandwich method to quantitate human TGF-β1 in samples. IL-6 analysis was conducted with the human IL-6 ELISA Kit from ELK Biotechnology (96 wells), used for serum samples, following the sandwich enzyme immunoassay method. The hs-CRP analysis was performed using the Beckman Coulter AU 5800 auto-analyzer, utilizing the immunoturbidimetry method.

Statistical analysis

All data were serialized and anonymized upon entry into MS Excel. Data were stored electronically with full confidentiality. Tests for normalization were checked, and appropriate transformations were applied if required. Continuous data were expressed in the form of Mean ± SD, and discrete data were expressed in the form of Counts (percentages). Comparisons were made using the Independent Samples' t-test and Chi-square test. Correlations were conducted through Pearson's r. Logistic regression was performed to find associations between the various studied parameters and account for various confounders. ROC curve analysis was performed to determine the diagnostic and predictive performance of the studied parameters. A p-value of less than 0.05 will be considered statistically significant. All analyses will be done using IBM SPSS v26.0 (IBM Corp., Armonk, NY, USA) and JASP v0.17.2.

## Results

The results were analyzed for demographic and anthropometric parameters, showing significant differences in systolic and diastolic blood pressure with a p-value of <0.001. No significant differences were observed in age, weight, height, and BMI, with a p-value of >0.05 among the three study groups. Significant differences were observed in systolic and diastolic BP: a) between normal non-pregnant women and preeclampsia women, with a p-value of <0.001, and b) between normal pregnant women and preeclampsia women, with a p-value of <0.001 (Appendix 1).

Regarding the hematological parameters, there was a significant difference among the groups in total hemoglobin (p = 0.001), total RBC count (p < 0.001), mean corpuscular volume (MCV) (p = 0.002), mean corpuscular hemoglobin (MCHB) (p = 0.003), red cell distribution width coefficient of variation (RDW CV) (p = 0.018), total leucocyte count (p < 0.001), neutrophils (p < 0.001), lymphocytes (p < 0.001), and eosinophils (p = 0.010). There was a significant difference between normal non-pregnant women and normal pregnant women in total hemoglobin (p = 0.002), total RBC count (p = 0.003), MCHB (p = 0.007), total leucocyte count (p = 0.002), neutrophils (p < 0.001), and lymphocytes (p < 0.001). There was also a significant difference between normal non-pregnant women and women with preeclampsia in total hemoglobin (p = 0.004), total RBC count (p < 0.001), MCV (p = 0.001), MCHB (p = 0.004), RDW CV (p = 0.017), total leucocyte count (p = 0.002), neutrophils (p < 0.001), and lymphocytes (p = 0.001). No significant difference exists between normal pregnant women and those with preeclampsia in hematological parameters (Appendix 2).

Regarding liver function tests (LFTs) and renal function tests (RFTs) parameters, there were significant differences among the groups in aspartate aminotransferase (AST) (p = 0.001), alanine aminotransferase (ALT) (p < 0.001), alkaline phosphatase (ALP) (p = 0.007), total protein (p < 0.001), albumin (p < 0.001), globulin (p < 0.001), creatinine (p = 0.042), and uric acid (p = 0.001). A significant difference was observed between normal women and normal pregnant women in total protein (p = 0.028), albumin (p < 0.001), and uric acid (p = 0.001). When comparing the parameters in normal women and PE cases, a significant difference existed in AST (p = 0.016), ALT (p = 0.008), ALP (p = 0.005), total protein (p < 0.001), albumin (p < 0.001), globulin (p < 0.001), and uric acid (p = 0.001). Between normal pregnant women and those with preeclampsia, there were significant differences in AST (p = 0.001), ALT (p < 0.001), total protein (p < 0.001), albumin (p = 0.047), and globulin (p < 0.001) (Appendix 3).

Results of flow cytometry analysis of T-regs among the groups show a significant difference in total lymphocytes (p = 0.001) and CD4+FOXP3+ T-regs (p < 0.001). Between NW/NP, a significant difference was seen in total lymphocytes (p = 0.001). Between NW/PE, a significant difference was seen in total lymphocytes (p = 0.005) and CD4+FOXP3+ T-regs (p < 0.001). Between NP/PE, significant differences were seen in CD4+FOXP3+ T-regs (p < 0.001) (Table 1).

**Table 1:** Flow cytometry analysis of T-regulatory cells in study groups with mean ± SD and significance. * : Statistical significance based on p-value of One-Way ANOVA test; a : Statistical significance based on p-value of post hoc test with Bonferroni. NW: Normal non-pregnant women in reproductive age group; NP: Normal pregnant group; PE: Preeclampsia group.

Parameters	Overall population (mean ± SD)	NW (mean ± SD)	NP (mean ± SD)	PE (mean ± SD)	Overall significance (P-value)	NW/NP (P-value)	NW/PE (P-value)	NP/PE (P-value)
Total Lymphocyte %	14.37 ± 10.94	25.86 ± 12.87	10.21 ± 6.98	13.02 ± 9.81	0.001*	0.001^a^	0.005^a^	1.00
CD3+CD4+ T-cells %	32.92 ± 6.34	35.43 ± 5.86	33.1 ± 6.26	31.47 ± 6.56	0.314	1.000	0.396	1.00
CD4+CD25+ T-regs %	4.68 ± 2.75	4.02 ± 1.52	5.54 ± 3.12	4.11 ± 2.69	0.211	0.526	1.000	0.349
CD4+FOXP3+ T-regs %	3.41 ± 1.64	3.99 ± 1.06	4.71 ± 0.92	1.73 ± 0.82	<0.001*	0.170	<0.001^a^	<0.001^a^
Absolute Total Lymphocytes (in no. of cells)	14373 ± 10939	25862 ± 12867	10210 ± 6975	13022 ± 9805	0.001*	0.001^a^	0.005^a^	1.00
Absolute CD3+CD4+ T-cells (in no. of cells)	10985 ± 2114	11822 ± 1955	11045 ± 2089	10502 ± 2189	0.314	1.000	0.396	1.00
Absolute CD4+CD25+ T-regs (in no. of cells)	1562 ± 918	1343 ± 509	1848 ± 1041	1371 ± 899	0.211	0.526	1.000	0.349
Absolute CD4+FOXP3+ T-regs (in no. of cells)	1137 ± 548	1333 ± 353	1573 ± 306	578 ± 274	<0.001*	0.170	<0.001^a^	<0.001^a^

Regarding inflammatory markers, a significant difference among the groups was observed in TGF-β1 (p = 0.023), IL-6 (p = 0.001), and hs-CRP (p = 0.024). No significant difference was observed between NW/NP. A significant difference was observed between NW and those with PE in TGF-β1 (p = 0.02) and IL-6 (p = 0.048). A significant difference was observed between NP and those with PE in IL-6 (p = 0.001) and hsCRP (p = 0.045) (Table 2).

**Table 2:** Analysis of Inflammatory markers in the study groups with mean ± SD and significance. * : Statistical significance based on p-value of One-Way ANOVA test; a: Statistical significance based on p-value of post hoc test with Bonferroni. NW: Normal non-pregnant women in reproductive age group; NP: Normal pregnant group; PE: Preeclampsia group; pg/ml: Picograms per milliliter; mg/l: Milligrams per liter.

Parameters	Overall population (Mean ± SD)	NW (Mean ± SD)	NP (Mean ± SD)	PE (Mean ± SD)	Overall significance (p-value)	NW/NP (p-value)	NW/PE (p-value)	NP/PE (p-value)
TGF-β1 (pg/ml)	5029.05 ± 2044.52	3569.09 ± 1535.97	5094.91 ± 2001.1	5693.16 ± 2016.82	0.023*	0.140	0.020^a^	0.994
IL-6 (pg/ml)	26.6 ± 19.08	21.76 ± 17.46	17.55 ± 13.37	38.05 ± 19.48	0.001*	1.000	0.048^a^	0.001^a^
hs-CRP (mg/l)	13.34 ± 33.29	1.76 ± 0.84	3.72 ± 2.33	28.75 ± 49.33	0.024*	1.000	0.094	0.045^a^

Urine routine and microscopy are used for the estimation of urine protein levels in normal pregnant women and those with preeclampsia. There were no cases of proteinuria in normal pregnant women and normal non-pregnant women. In the PE group, 13 cases had urine protein levels of approximately 30 mg/dl, and three cases had urine protein levels of approximately 100 mg/dl (Table 3).

**Table 3:** Number of participants in each group with protein in urine. NW: Normal non-pregnant women in reproductive age group; NP: Normal pregnant group; PE: Preeclampsia group; mg/dl: Milligrams per decilitre.

Parameter	Protein in urine (mg/dl)	Overall population	NW	NP	PE
Urine protein	No proteinuria	34	10	20	4
	30 mg/dL	13	0	0	13
	100 mg/dL	3	0	0	3
	300 mg/dL	0	0	0	0

**Figure 1:**
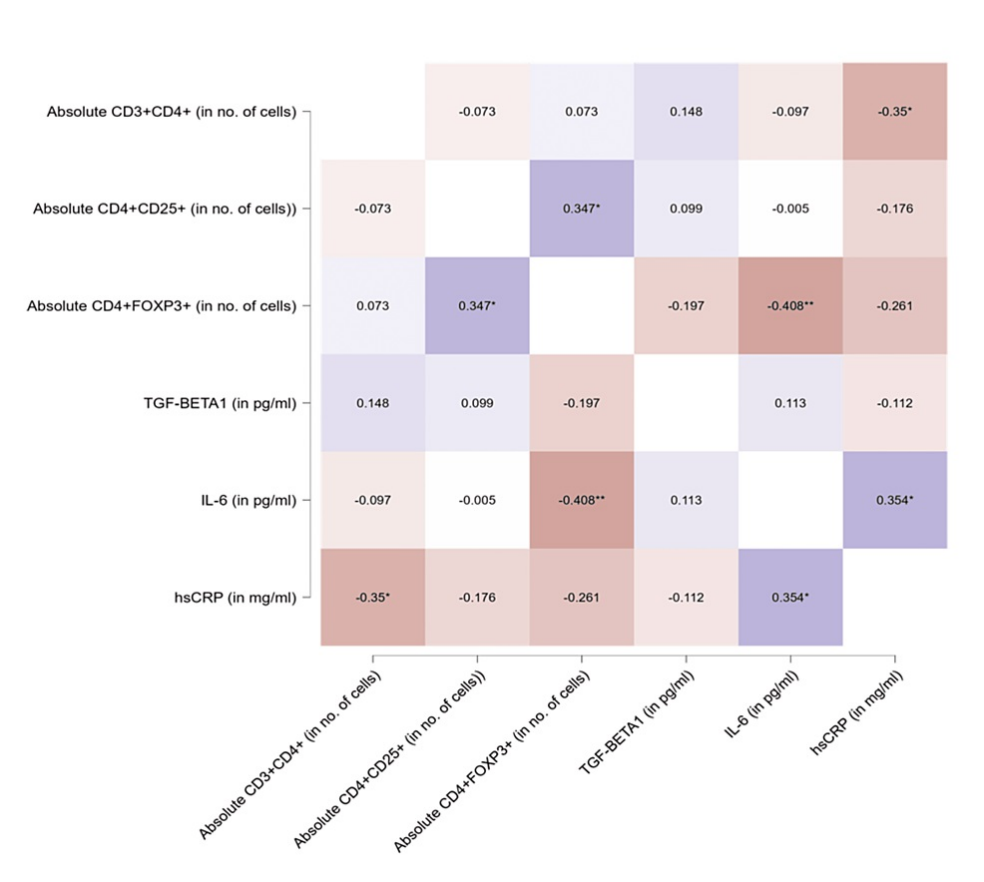
Correlation heatmap by Pearson's r for T-regulatory cells and inflammatory markers. * P-value <0.05, ** p-value <0.01, *** p-value = 0.001. TGF-β1: Transforming growth factor-beta1, IL-6: Interleukin-6, and hsCRP: high sensitive C-Reactive protein.

Figure 1 shows a correlation heatmap by Pearson's r between the T-regulatory cells and inflammatory markers. A significant positive correlation was observed between (a) absolute CD4+CD25+ and absolute CD4+FOXP3+ (r = 0.347, p < 0.05), (b) IL-6 and hs-CRP (r = 0.354, p < 0.05). A significant negative correlation was observed between (a) absolute CD3+CD4+ and hs-CRP (r = -0.35, p < 0.05), (b) absolute CD4+FOXP3+ and IL-6 (r = -0.408, p < 0.01).

**Figure 2:**
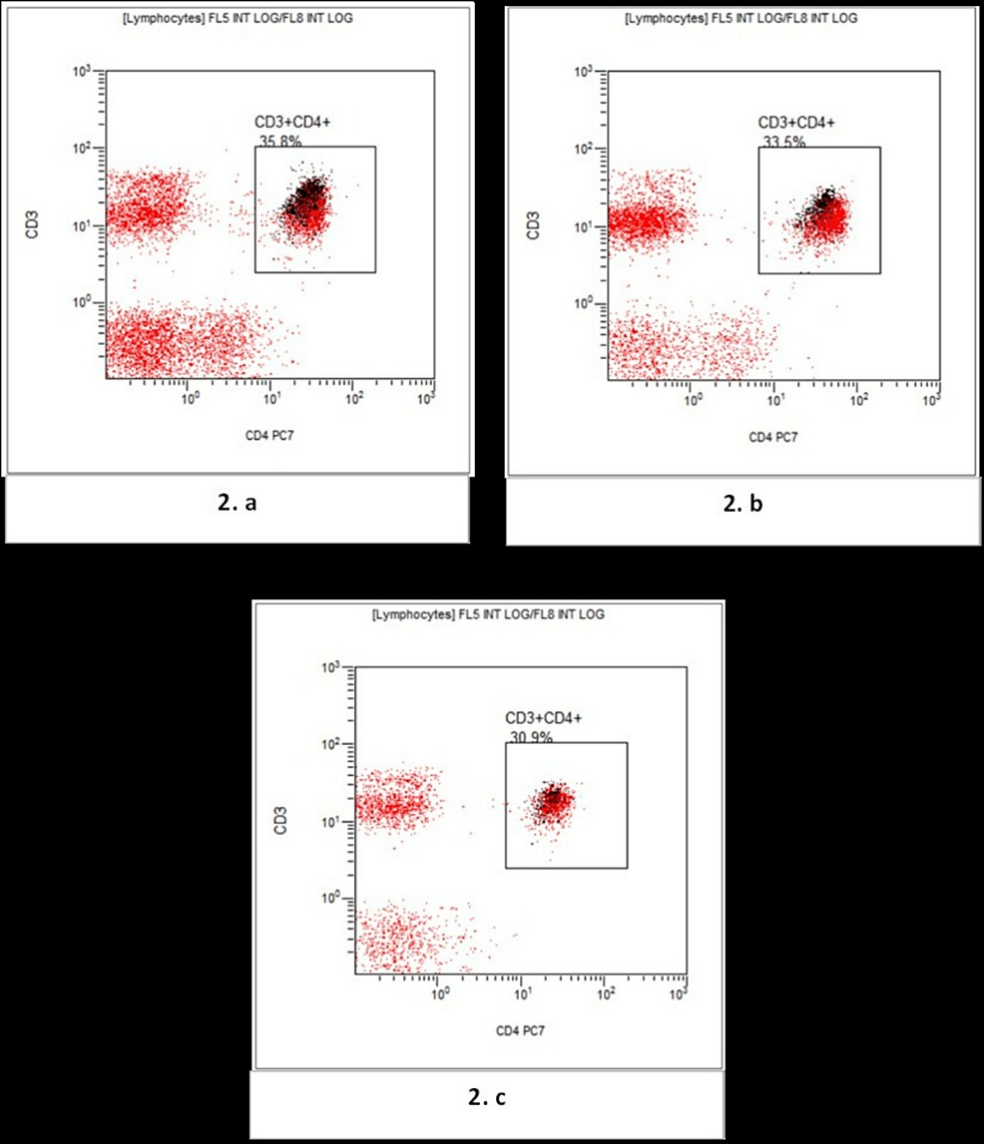
Flow cytometry flow-page showing the gating of total CD3+CD4+T-Lymphocytes. (a) Normal non-pregnant reproductive age group women; (b) Normal pregnant; (c) Preeclampsia.

Figure 2 shows a comparative flow cytometry analysis showing levels of CD3+CD4+ T-lymphocytes in the study groups.

**Figure 3:**
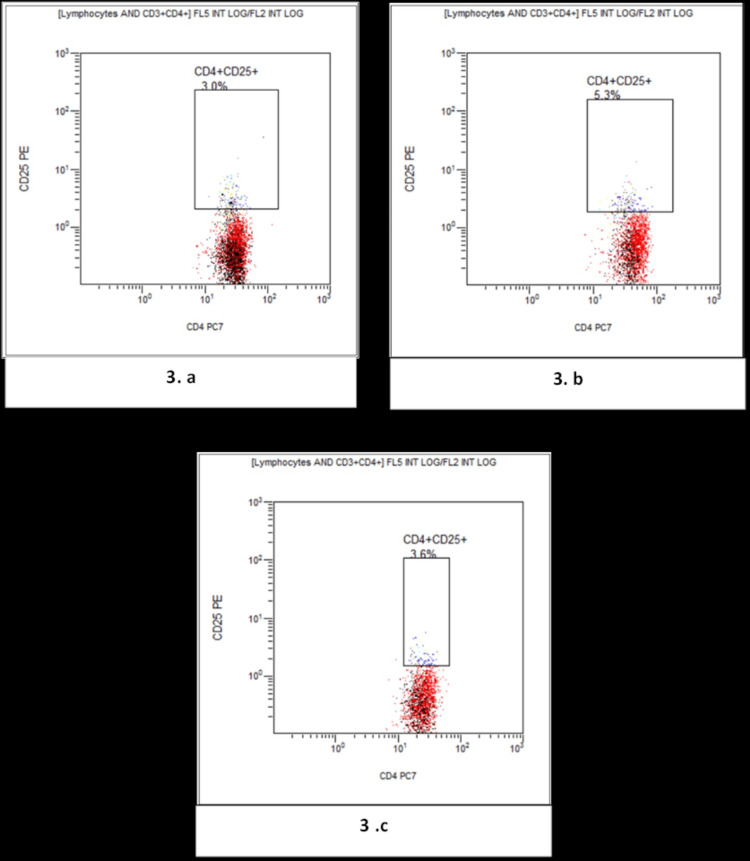
Flow cytometry flow-page showing the gating of CD4+CD25+ T-regulatory cells. (a) Normal non-pregnant reproductive age group women; (b) Normal Pregnant; (c) Preeclampsia.

Figure 3 presents a comparative flow cytometry analysis showing levels of CD4+CD25+ T-regs in the study groups.

**Figure 4:**
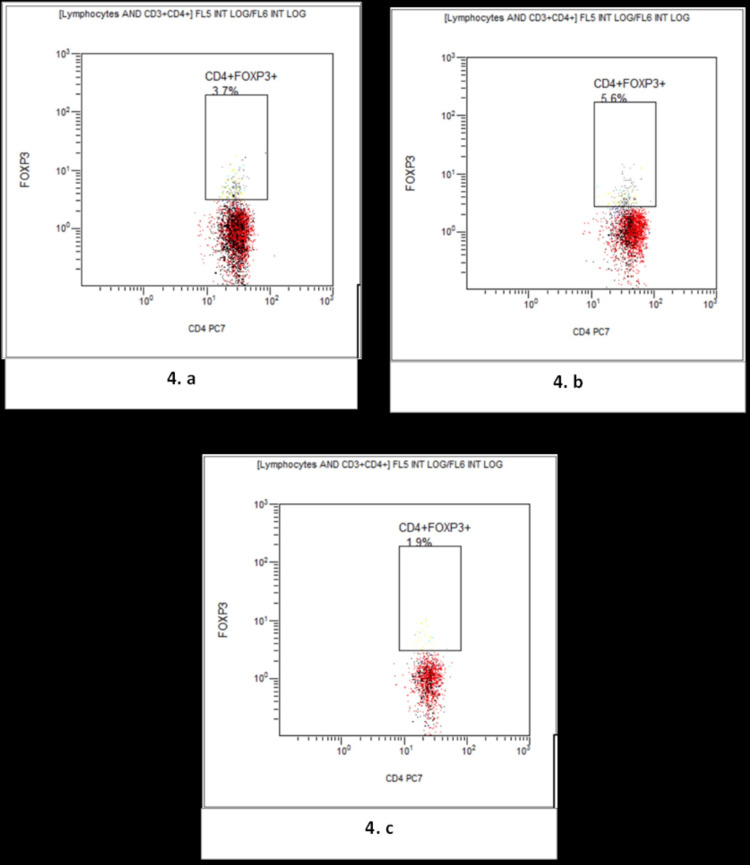
Flow cytometry flow-page showing the gating of total CD4+FOXP3+ T-regulatory cells. (a) Normal non-pregnant reproductive age group women; (b) Normal pregnant; (c) Preeclampsia.

Figure 4 presents a comparative flow cytometry analysis showing levels of CD4+FOXP3+ T-regs in the study groups.

**Figure 5:**
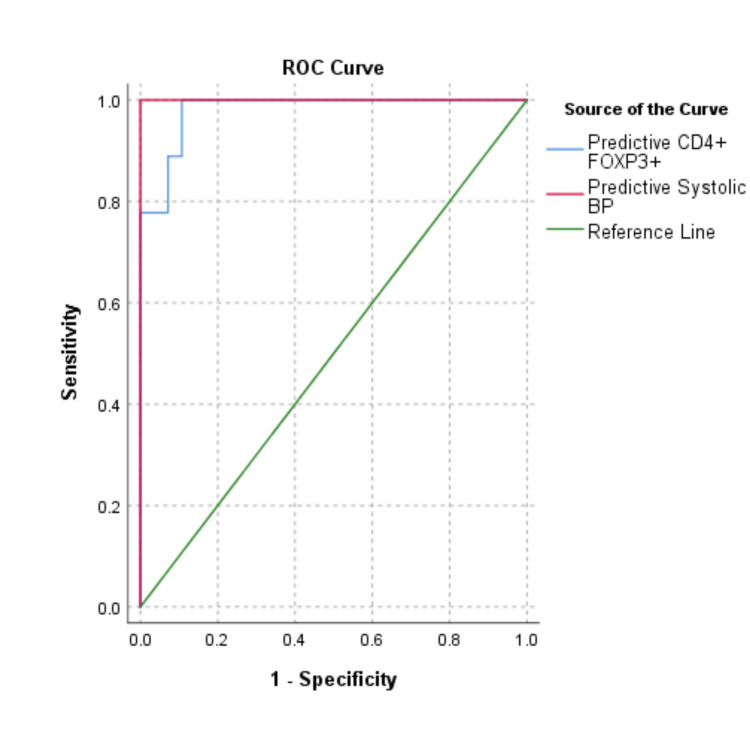
ROC curve analysis to determine the diagnostic power of systolic BP and CD4+FOXP3+ T-regs. ROC: Receiver operating characteristic; BP: Blood pressure; T-regs: T-regulatory cells.

Logistic regression was performed to identify parameters significantly associated with predicting preeclampsia. When all the studied parameters were considered, only systolic BP was found to be significant (B = 2.358, Exp(B) = 10.567, Cox & Snell R2 = 0.750, p < 0.001). Similarly, when only immunological parameters were considered, CD4+ FOXP3+ % was significant (B = -0.012, Exp(B) = 0.989, Cox & Snell R2 = 0.641, p < 0.001) in detecting preeclampsia. These adjusted predictive probabilities exhibited very high diagnostic potential. Systolic BP had an area under the curve (AUC) of 1.00 (p < 0.001), and CD4+ FOXP3+ % had an AUC of 0.980 (p < 0.001). However, the paired sample AUC difference for both these curves was not significant (AUC difference = 0.020, z = 1.345, p = 0.179) (Figure 5).

## Discussion

The focus of this study is to evaluate the T-regs in the PE group compared to the NP group and NW group and to correlate the T-regs with inflammatory markers like TGF-β1, IL-6, and hs-CRP in the pathophysiology and for early diagnosis of preeclampsia. A total of 50 subjects were recruited for this study, out of which 20 subjects were PE cases, 20 were normal pregnant women, and 10 were normal non-pregnant reproductive age group women. The subjects were recruited according to inclusion and exclusion criteria from OPD and IPD cases from the Department of Obstetrics and Gynaecology at AIIMS, Bhubaneswar. The anthropometric measurements show no significant difference in age, height, weight, BMI, waist, and hip circumference. The absence of significant differences among the study groups strengthens the findings of this study. This indicates that the observed variations in T-regs and inflammatory markers are likely attributed to the specific conditions under investigation, namely PE. Controlling for these potential confounding variables enhances the validity and reliability of the study's results, providing valuable insights into the immunological and inflammatory changes associated with preeclampsia. There is a significant difference in both systolic and diastolic blood pressure between normal non-pregnant women and those with preeclampsia (p < 0.001), as well as between normal pregnant women and those with preeclampsia (p < 0.001) (Appendix 1). T-regs are a distinct subset pool of CD4+ lymphocytes that control the immune system's innate and adaptive responses to self-antigens, infectious pathogens, cancer, commensal microbiota, and several other allergens [11]. T-regs play a vital role in the mechanisms of maternal-fetal tolerance, contributing to an effective immunological role in protecting the allogenic fetus during pregnancy and preventing pregnancy-related complications [12]. Our study identified that the levels of CD4+CD25+ T-regs were lower in PE than in normal pregnancy, but the difference was non-significant (p = 0.349) (Table 1). FOXP3 is considered the most specific marker for T-regs. In this study, we observed that the levels of CD4+FOXP3+ T-regs in preeclampsia were significantly reduced when compared to both normal pregnant women (p < 0.001) and normal non-pregnant women (p < 0.001). Toldi G et al. investigated the proportion of T-regs and their cellular network in preeclamptic women, and they have seen that the proportion of T-regs and that of naive CD4+CD45RA+ cells were lower in preeclamptic than in control women. The researchers proposed a few possible explanations for decreased T-regs based on their findings: (a) impaired T-reg development; (b) decreased survival of T-regs; (c) enhanced apoptosis; and (d) dysregulated immune system by imbalance cytokine production [13]. Prins JR et al. hypothesized that levels of T-regs were decreased in preeclamptic patients. Women with preeclampsia had significantly lower percentages of CD4+FOXP3+ T-regs and concluded that a deficiency of T-regs may play a role in the development of PE [14]. Our study corroborated the findings of the observations, as mentioned earlier.

Care AS et al. demonstrated that T-regs play an essential role in modulating uterine artery function during pregnancy, and they identified Treg-cell control of maternal vascular function as a key mechanism in the normal development of the fetus and placenta [15]. According to research by Teles A et al., maternal-fetal tolerance develops at the molecular level through the identification of fetal antigens by the mother's immune system. This identification triggers a series of immunological mechanisms to control subsequent maternal antigen-specific reactions. In these pathways of maternal-fetal immunological tolerance, T-regs play a key role not only during pregnancy but also in successful implantation and even before fertilization [12].

Zhao JX et al. demonstrated that the CD4+CD25+T-regs frequencies in mice with ovariectomized ovaries are unaffected by estrogen, progesterone, or hormone alone or combined. These findings imply that fetal alloantigen is the cause of the rise in T-regs throughout pregnancy and that the expansion of the T-reg population is crucial for the allogeneic fetus to avoid the mother's immune attack [16]. Gomez-Lopez N et al., 2020, demonstrated that the T-regs have recently been shown to play a crucial role in the development of the fetus in late pregnancy, as it has been shown that their loss causes preterm birth, fetal growth restriction (FGR), bradycardia, and bradycardia without affecting uterine or umbilical arterial blood flow. Additionally, it has been shown that the loss of T-regs leads to the dysregulation of numerous cellular and developmental processes in the placenta [17]. According to our results, the levels of T-regs are reduced, which may lead to an imbalance in immune regulation, potentially contributing to the development of PE.

The levels of TGF-β1 in PE have been a subject of investigation, with findings varying across studies. Some studies have reported elevated levels of TGF-β1 in preeclampsia cases compared to healthy pregnant women. Increased TGF-β1 levels have been associated with endothelial dysfunction and increased vascular resistance, which are characteristic features of preeclampsia [18]. Conversely, other studies have found decreased levels of TGF-β1 in preeclampsia cases compared to normal pregnancies. These low TGF-β1 levels have been linked with impaired immune regulation and heightened inflammation, which are also connected to preeclampsia [19]. However, some studies have not observed significant differences in TGF-β1 levels between preeclampsia cases and healthy pregnant women [20]. These variations could be attributed to factors such as differences in study populations, methodologies, and the heterogeneity of preeclampsia. Thus, more research is needed to clarify the function of TGF-β1 and its potential implications in preeclampsia pathogenesis. In our study, we found that TGF-β1 levels in preeclampsia were significantly higher than in normal women (p = 0.020), and when compared with normal pregnant women, the levels were higher but not significant (p = 0.994). This finding aligns with documentation in various other studies (Table 2). Peraçoli MT et al. determined the plasma concentration of TGF-β1 in normal pregnancy and preeclampsia women in the third trimester and found that active TGF-β1 levels were significantly higher in preeclamptic women (10.41 ± 2.07 ng/mL) compared with normal pregnant women (7.01 ± 3.29 ng/mL) [18]. Wang X et al. conducted a meta-analysis to identify differences in circulating TGF-β1 levels between preeclampsia and normal pregnancies. They found that women with preeclampsia had higher circulating TGF-β1 levels during active disease compared to normotensive controls (SMD, 0.94 [95% CI, 0.52 to 1.35]; P = 0.000). Circulating TGF-β1 levels were higher in both early-onset/severe and late-onset/mild types of preeclampsia [21]. Wang Y et al. studied the expression of TGF-β1, endothelium-selectin (E-selectin), and vascular cell adhesion molecule-1 (VCAM-1) in the placenta of preeclampsia patients. They found that TGF-β1 levels were significantly higher in the preeclampsia group than the control group, while E-selectin and VCAM-1 levels were significantly lower [22]. However, several other studies have diverged from our observation of TGF-β1. For instance, Yusrawati et al. compared the mean difference of TGF-β1 between preeclampsia and normal pregnancy, finding that the mean difference was lower in the preeclampsia group (2.02 ± 0.99 ng/mL vs. 3.24 ± 2.67 ng/mL; p<0.05) [19]. Huber A et al. also reported no difference in serum TGF-β1 levels between preeclampsia and healthy pregnant women, with levels not being associated with disease severity or correlated with clinical maternal or fetal parameters [20].

These varied results suggest that pregnancy is a condition associated with higher levels of anti-angiogenic and pro-inflammatory factors compared to the non-pregnant state, and that PE is characterized by an imbalance of these factors in the maternal circulation [23].

Regarding IL-6, we found that the levels were higher in preeclampsia when compared with normal pregnant women (p = 0.01) and normal non-pregnant women (p = 0.048). Our results are supported by observations made in various other studies (Table 2).

Aggarwal R et al. studied the expression of pro-inflammatory (TNF-α, IL-6) and anti-inflammatory (IL-4, IL-10) cytokines in the serum and placenta of preeclamptic pregnant women. They observed that, in comparison to controls, preeclamptic cases had significantly higher levels of the pro-inflammatory cytokines TNF-α and IL-6, whereas lower levels of the anti-inflammatory cytokines IL-4 and IL-10 were present [24]. Teran E et al. investigated the concentrations of inflammatory markers in non-pregnant women, normal pregnant women, and women with pre-eclampsia. They observed that the IL-6 levels were significantly higher in pre-eclamptic women compared with normal pregnant and normal non-pregnant women. TNF-α and CRP levels were also significantly higher in preeclampsia than in the control group and normal pregnancy [25]. Afshari JT et al. determined the role of Interleukin-6 (IL-6) and Tumour Necrosis Factor-alpha (TNF-α), markers of immune activation and endothelial dysfunction, in patients with preeclampsia. They observed that IL-6 levels were significantly higher in preeclamptic women compared to normal pregnant women (p = 0.02), and regarding TNF-α, there was no significant change in concentration in preeclamptic women compared to normal pregnant women (p > 0.1) [26].

In our study concerning hsCRP, we observed that the levels in preeclampsia were significantly increased when compared with normal pregnant women (p = 0.045) and normal non-pregnant women (p = 0.094) (Table 2).

Kara AE et al. evaluated the serum levels of hs-CRP, sialic acid (SA), and IL-6 in women with PE and intrauterine growth restriction (IUGR), and compared them to healthy pregnancies. They found that the mean levels of hsCRP, sialic acid, and interleukin-6 were higher in the PE and IUGR groups than in the control group, but these differences were statistically insignificant (p > 0.05). Additionally, no significance was observed between these inflammatory markers (p > 0.05) [27]. Lumbreras-Marquez MI et al. reported maternal and umbilical vein levels of procalcitonin (PCT) in patients with PE compared to controls. They also assessed IL-6 and hs-CRP as secondary goals. PCT, hs-CRP, and IL-6 maternal plasma and serum concentrations were greater in the PE group. PCT and hs-CRP values in umbilical venous samples were greater in the PE group than in the controls. There was no difference in umbilical venous IL-6 concentrations between the PE and control groups. A positive correlation was observed for both PCT and hs-CRP with MAP in maternal and umbilical venous samples. However, there was no correlation between IL-6 and MAP in maternal or umbilical venous samples [28].

The results of our study demonstrate that PE is associated with decreased T-regs and increased TGF-β1, IL-6, and hs-CRP levels. A significant positive Pearson's correlation was observed between a) absolute CD4+CD25+ counts and absolute CD4+FOXP3+ counts (r = 0.347, p < 0.05), and b) IL-6 and hs-CRP (r = 0.354, p < 0.05). Additionally, a significant negative correlation was observed between a) absolute CD3+CD4+ counts and hs-CRP (r = -0.35, p < 0.05), and b) absolute CD4+FOXP3+ counts and IL-6 (r = -0.408, p < 0.01) (Figure 1). These correlations suggest that T-regs (CD4+FOXP3+) decrease with an increase in pro-inflammatory cytokines (such as IL-6) or vice versa, indicating reduced immune regulation in pro-inflammatory conditions like PE.

These results are consistent with earlier studies that indicate immunological dysregulation and inflammation are key factors in the etiology of PE. T-regs play a crucial role in immune response regulation and are in charge of preserving immunological tolerance and limiting excessive inflammation. The decrease in T-regs observed in preeclampsia may disrupt this immunological balance, potentially leading to an excessive inflammatory response. The rise in TGF-β1 concentrations is consistent with the cytokine's function as an immunoregulatory agent. Increased TGF-β1 levels in PE might be an attempt to promote immune suppression and tissue remodeling to balance the inflammatory environment. It is crucial to remember that additional research is necessary to determine the precise pathways through which TGF-β1 contributes to the pathophysiology of PE. Since IL-6 is a pro-inflammatory cytokine, the rise in IL-6 levels is a sign of inflammation. In PE, elevated IL-6 levels have been linked to endothelial dysfunction and poor placental development. The increased production of IL-6, which exacerbates the inflammatory response and further jeopardizes vascular function, may be caused by the dysregulation of T-regs that we have found in this study. The rise in hs-CRP levels is a sign of widespread inflammation, which aligns with PE's inflammatory nature. Elevated hs-CRP levels have been associated with increased cardiovascular risk and endothelial dysfunction. As T-regs are responsible for suppressing excessive immune activation and inflammation, their decreased levels in preeclampsia may contribute to this systemic inflammation.

Limitations

Trimester evaluation could not be done due to fewer early-onset PE cases in the study group. Follow-up studies of registered cases could have thrown more light on understanding the role of T-regs in the progression of the disease and maternal and fetal outcomes.

## Conclusions

Our study unequivocally establishes a strong association between PE and reduced T-regs and is accompanied by elevated levels of pro-inflammatory cytokines (TGF-β1, IL-6, and hs-CRP). These findings suggest that monitoring these parameters could serve as a biomarker panel for early PE detection and identifying at-risk individuals. The reciprocal correlation observed between decreased T-regs and increased pro-inflammatory cytokines underscores a compromised immune regulation in the context of PE, indicating its potential role in the pathogenesis and severity of this complex condition. These findings open new approaches for research and therapeutic interventions, aiming to restore immune balance and assist in managing PE. Personalized care based on immune and inflammatory profiles could optimize maternal and fetal outcomes in PE.

## Appendices

Appendix 1

**Table 4:** Demographic and anthropometric parameters, characteristics, Mean ± SD, and significance of the study groups. *: Statistical significance based on p-value of one-way ANOVA test;  (a): Statistical significance based on p-value of post hoc test with Bonferroni; (b) Only two active groups so p-value is based on Independent Samples’ t-test; (c): p-value based on Chi-Square test. NW: Normal non-pregnant women in reproductive age group; NP: Normal pregnant group; PE: Preeclampsia group.

Parameters		Overall Population (Mean ± SD)	NW (Mean ± SD)	NP (Mean ± SD)	PE (Mean ± SD)	Overall Significance	NW/NP (p-value)	NW/PE (p-value)	NP/PE (p-value)
Age (in years)		28.08 ± 3.76	28.9 ± 1.52	27.7 ± 3.6	28.05 ± 4.68	0.720	1.000	1.000	1.00
Gestational Age (in weeks)		34.97 ± 4.34	-	34.35 ± 4.93	35.59 ± 3.68	0.373^ b^			
Primi-Gravida	No	23	-	13	10				0.262 ^c^
	Yes	17	-	7	10				
<34 Weeks	No	27	-	11	16				0.888 ^c^
	Yes	13	-	9	4				
Weight Gained (in kg)		9.72 ± 2.13	-	9.79 ± 2.46	9.65 ± 1.81	0.839^ b^	-	-	0.839^ b^
Weight (in kg)		56.68 ± 8.46	58.7 ± 8.59	56.75 ± 6.86	55.6 ± 9.96	0.648	1.000	1.000	1.00
Height (in cm)		152.98 ± 6.72	155.85 ± 5.82	153.43 ± 5.69	151.1 ± 7.72	0.177	1.000	0.210	0.815
BMI (in kg/m2)		24.22 ± 3.18	24.16 ± 3.12	24.07 ± 2.11	24.4 ± 4.12	0.948	1.000	1.000	1.00
Waist To Hip Ratio		0.84 ± 0.04	0.83 ± 0.04	0.85 ± 0.03	0.84 ± 0.04	0.639	1.000	1.000	1.00
Systolic BP (in mm of Hg)		129.94 ± 18.19	117.3 ± 4.16	116.15 ± 5.83	150.05 ± 10.07	<0.001*	1.000	<0.001^a^	<0.001^a^
Diastolic BP (in mm of Hg)		87.54 ± 14.26	80.1 ± 2.56	76 ± 4.84	102.8 ± 9.13	<0.001*	0.357	<0.001^a^	<0.001^a^

Appendix 2

**Table 5:** Complete blood count characteristics of the study groups with mean ± SD and significance. *: Statistical significance based on p-value of one-way ANOVA test,  a : Statistical significance based on p-value of post hoc test with Bonferroni. NW: Normal non-pregnant women in reproductive age group; NP: normal pregnant group; PE: Preeclampsia group; MCV: Mean corpuscular volume; MCHB: Mean corpuscular hemoglobin; RDW-CV: Red cell distribution width coefficient of variation.

Parameters	Overall Population (Mean ± SD)	NW (p-value)	NP (p-value)	PE (p-value)	Overall Significance	NW/NP (p-value)	NW/PE (p-value)	NP/PE (p-value)
Total Haemoglobin (in g/dL)	11.36 ± 1.16	12.5 ± 0.86	11.01 ± 0.75	11.15 ± 1.31	0.001*	0.002^a^	0.004^a^	1.00
Total RBC Count (in million/mm3)	4.2 ± 0.57	4.82 ± 0.59	4.16 ± 0.39	3.92 ± 0.5	<0.001*	0.003^a^	<0.001^a^	0.359
PCV (in %)	33.77 ± 5.29	31.29 ± 9.08	35 ± 3.2	33.78 ± 4.26	0.197	0.220	0.675	1.00
MCV (in fL)	86.71 ± 6.68	80.92 ± 7.68	86.63 ± 5.83	89.69 ± 5.09	0.002*	0.051	0.001^a^	0.334
MCHB (in pg)	29.8 ± 2.3	31.92 ± 1.66	29.35 ± 2.57	29.18 ± 1.66	0.003*	0.007^a^	0.004^a^	1.00
MCHB Concentration (g/dL)	32.46 ± 0.99	32.28 ± 1.3	32.48 ± 0.93	32.54 ± 0.92	0.805	1.000	1.000	1.00
RDW CV (in %)	15.2 ± 1.28	16 ± 1.09	15.34 ± 1.09	14.66 ± 1.33	0.018*	0.473	0.017^a^	0.242
Total Leucocyte Count (in 1000/mm3)	9.07 ± 3.66	5.28 ± 0.86	9.76 ± 3.44	10.27 ± 3.58	<0.001*	0.002^a^	0.001^a^	1.00
Neutrophil %	72.16 ± 6.27	64.83 ± 5.59	73.25 ± 3.45	74.73 ± 6.19	<0.001*	<0.001^a^	<0.001^a^	1.00
Lymphocytes %	21.59 ± 4.91	26.98 ± 2.8	20.05 ± 3.21	20.44 ± 5.39	<0.001*	<0.001^a^	0.001^a^	1.00
Eosinophils %	2.37 ± 1.31	2.67 ± 1.03	2.89 ± 1.08	1.71 ± 1.41	0.010*	1.000	0.138	0.11
Monocytes %	4 ± 1.56	4.45 ± 1.79	4.14 ± 1.72	3.65 ± 1.23	0.377	1.000	0.574	0.990
Basophils %	0.54 ± 0.33	0.74 ± 0.28	0.48 ± 0.3	0.51 ± 0.36	0.106	0.132	0.203	1.00
Total Platelet Count (in 1000/mm3)	212.86 ± 61.09	226.9 ± 57.32	194.1 ± 32.62	224.6 ± 79.9	0.210	0.501	1.000	0.350

Appendix 3 

**Table 6:** Liver function tests and renal function tests characteristics among the study groups with mean ± SD and significance. *: Statistical significance based on p-value of one-way ANOVA test, a : Statistical significance based on p-value of post hoc test with Bonferroni. NW: Normal non-pregnant women in reproductive age group; NP: Normal pregnant group; PE: Preeclampsia group.

Parameters	Overall Population (Mean ± SD)	NW (p-value)	NP (p-value)	PE (p-value)	Overall Significance (p-value)	NW/NP (p-value)	NW/PE (p-value)	NP/PE (p-value)
Total Bilirubin (in mg/dL)	0.49 ± 0.19	0.5 ± 0.21	0.51 ± 0.18	0.48 ± 0.2	0.877	1.000	1.000	1.00
Direct Bilirubin (in mg/dL)	0.13 ± 0.05	0.14 ± 0.05	0.13 ± 0.04	0.13 ± 0.04	0.655	1.000	1.000	1.00
Indirect Bilirubin (in mg/dL)	0.36 ± 0.15	0.36 ± 0.16	0.38 ± 0.15	0.35 ± 0.16	0.880	1.000	1.000	1.00
AST (in IU/L)	37.43 ± 21.47	29.3 ± 8.74	28.27 ± 7.14	50.65 ± 28.18	0.001*	1.000	0.016^a^	0.001^a^
ALT (in IU/L)	36.48 ± 23.8	27.4 ± 7.71	25.22 ± 10.91	52.29 ± 29.56	<0.001*	1.000	0.008^a^	<0.001^a^
ALP (in IU/L)	125.46 ± 72.08	68.9 ± 32.49	125.45 ± 31.55	153.75 ± 96.84	0.007*	0.098	0.005^a^	0.551
Total Protein (in g/dL)	6.53 ± 0.71	7.27 ± 0.73	6.73 ± 0.4	5.97 ± 0.49	<0.001*	0.028	<0.001^a^	<0.001^a^
Albumin (in g/dL)	3.49 ± 0.32	3.91 ± 0.22	3.49 ± 0.19	3.3 ± 0.28	<0.001*	<0.001^a^	<0.001^a^	0.047^a^
Globulin (in g/dL)	3.06 ± 0.5	3.47 ± 0.4	3.25 ± 0.29	2.67 ± 0.45	<0.001*	0.403	<0.001^a^	<0.001^a^
Albumin to Globulin Ratio	1.15 ± 0.23	1.06 ± 0.31	1.11 ± 0.14	1.24 ± 0.24	0.077	1.000	0.140	0.212
Urea (in mg/dL)	29.84 ± 14.56	27.8 ± 10.21	25.45 ± 11.07	35.25 ± 17.94	0.090	1.000	0.540	0.10
Creatinine (in mg/dL)	0.85 ± 0.45	0.7 ± 0.29	0.73 ± 0.21	1.05 ± 0.61	0.042*	1.000	0.129	0.080
Uric Acid (in mg/dL)	5.18 ± 1.81	3.31 ± 1.68	5.63 ± 1.78	5.67 ± 1.28	0.001*	0.001^a^	0.001^a^	1.00
Sodium (in mEq/L)	137.42 ± 4.9	138.9 ± 3.11	138.1 ± 3.42	136 ± 6.48	0.230	1.000	0.389	0.533
Potassium (in mEq/L)	4.06 ± 0.34	3.88 ± 0.24	4.08 ± 0.24	4.13 ± 0.43	0.150	0.364	0.171	1.00
Chloride (in mEq/L)	100.66 ± 2.94	99.9 ± 3.07	100.7 ± 2.43	101 ± 3.39	0.634	1.000	1.000	1.00
